# Rehabilitation in Pediatric Heart Failure and Heart Transplant

**DOI:** 10.3389/fped.2021.674156

**Published:** 2021-05-19

**Authors:** Ana Ubeda Tikkanen, Emily Berry, Erin LeCount, Katherine Engstler, Meredith Sager, Paul Esteso

**Affiliations:** ^1^Department of Pediatric Rehabilitation, Spaulding Rehabilitation Hospital, Boston, MA, United States; ^2^Department of Cardiac Surgery, Boston Children’s Hospital, Boston, MA, United States; ^3^Department of Orthopedic Surgery, Boston Children’s Hospital, Boston, MA, United States; ^4^Department of Physical Medicine and Rehabilitation, Harvard Medical School, Boston, MA, United States; ^5^Department of Physical Therapy and Occupational Therapy Services, Boston Children’s Hospital, Boston, MA, United States; ^6^Department of Otolaryngology and Communication Enhancement, Boston Children’s Hospital, Boston, MA, United States; ^7^Augmentative Communication Program, Boston Children’s Hospital, Boston, MA, United States; ^8^Department of Cardiology, Boston Children’s Hospital, Boston, MA, United States; ^9^Department of Pediatrics, Harvard Medical School, Boston, MA, United States

**Keywords:** heart failure, heart transplant, rehabilitation, function, pediatrics, physical therapy, speech therapy, feeding therapy

## Abstract

Survival of pediatric patients with heart failure has improved due to medical and surgical advances over the past decades. The complexity of pediatric heart transplant patients has increased as medical and surgical management for patients with congenital heart disease continues to improve. Quality of life in patients with heart failure and transplant might be affected by the impact on functional status that heart failure, heart failure complications or treatment might have. Functional areas affected might be motor, exercise capacity, feeding, speech and/or cognition. The goal of rehabilitation is to enhance and restore functional ability and quality of life to those with physical impairments or disabilities. Some of these rehabilitation interventions such as exercise training have been extensively evaluated in adults with heart failure. Literature in the pediatric population is limited yet promising. The use of additional rehabilitation interventions geared toward specific complications experienced by patients with heart failure or heart transplant are potentially helpful. The use of individualized multidisciplinary rehabilitation program that includes medical management, rehabilitation equipment and the use of physical, occupational, speech and feeding therapies can help improve the quality of life of patients with heart failure and transplant.

## Introduction

The development of advanced heart failure management strategies and associated need for heart transplantation in pediatric patients is increasing survival in pediatric patients with congenital heart disease. This increase in survival will often be accompanied by suboptimal quality of life and functional outcomes. Multidisciplinary rehabilitation programs will be critical to improve patient outcomes.

### Definition of Heart Failure

Heart failure is a complex clinical syndrome of inappropriate oxygen delivery secondary to a number of physiologic derangements. These typically include congenital or acquired heart disease with volume or pressure overload, inappropriate systolic function, inappropriate diastolic function or inappropriate blood oxygen carrying capacity (1). Advanced heart failure is that in which clinical symptoms cannot be readily managed with medications and consideration of advanced therapies including ventricular assist devices (VAD) and heart transplantation are indicated.

### Incidence and Prevalence of Pediatric Heart Failure

Precise documentation of the incidence and prevalence of heart failure in pediatric patients has been elusive, though significant demographic and geographic differences in epidemiology are evident. Incidence has been reported to range from 0.87/100,000 in the UK and Ireland while 7.4/100,000 in Taiwan (2). The underlying cause is frequently associated with congenital heart disease (CHD) or genetic cardiomyopathy in the USA while a majority of pediatric patients with heart failure in Nigeria have rheumatic heart disease (2). Prevalence has not been well-defined in the United States; however, it has been reported that more than 14,000 children are hospitalized yearly for heart failure (3, 4). The development of advanced heart failure and associated complications are closely tied to the underlying etiology of the heart failure. In children with dilated cardiomyopathy (DCM) cumulative incidence of recovery at 10 years after diagnosis is >60% for patient with myocarditis, ~35% in patients with familial DCM and <20% in children with neuromuscular disorders while the corresponding rates of transplantation and death are each ~10% for myocarditis, 30 and 10% respectively for familial DCM and ~20 and 60% respectively for children with neuromuscular disorders (5).

### Incidence of Heart Transplant Listing in Pediatric Patients

A recent query from our group of the Organ Procurement and Transplantation Network (OPTN), the American governmental entity overseeing transplantation, noted 1,789 pediatric heart transplant candidate (<18 years old) listings during a 33-month period between 2016 and 2019 (6). This represents ~650 pediatric heart transplant patients listed per year. Though the number of pediatric heart transplants in the US has increased yearly there remains a significant deficit relative to listings. The number of pediatric heart transplants undertaken, according to OPTN data, were 431 in 2017, 468 in 2018, 507 in 2019 and 461 in 2020. In addition to normal year-to-year variation there is a potential for the COVID-19 pandemic to have affected the 2020 pediatric heart transplant numbers, which was well documented in adult heart transplantation (7). Notably, COVID-19 infection and multisystem inflammatory syndrome in children (MIS-C) have also been reported to lead to cardiac dysfunction in pediatric patients (8, 9). Thus, the effect of the COVID-19 pandemic on the development of heart failure, need for transplantation and availability of heart transplant donors and resources has yet to be fully realized.

### Evolving Complexity of Heart Failure

The epidemiology of pediatric heart failure and heart transplantation in the United States and Europe has necessarily reflected the parallel surgical management of CHD. As reported in the most recent report from the International Society of Heart and Lung Transplantation (ISHLT), patients <1 year of age undergoing heart transplantation had congenital heart disease 72% of the time between 1988 and 2004, while only 57% did between 2010 and 2018 (10). This is in contrast to children 11–17 years of age who were transplanted with congenital heart disease at similar rates, nearly 25%, in the corresponding eras (10). Additionally, the complexity of patients with CHD has increased. A longitudinal report from pediatric heart transplantations undertaken at Stanford University, over a 40-year period 1974–2014, describes a significant increase over time in the proportion of patients with CHD, specifically those with single ventricle physiology, as well as VAD support (11).

### Outcomes for Single Ventricle Patients Palliated to Fontan

It is notable that though the staged palliation of patients to Fontan circulation has resulted in improved survival for these patients (12) they continue to experience advanced heart failure at increased rates (13). Retrospective analysis of the Single Ventricle Reconstruction Trial, a prospective study of infants undergoing the Norwood procedure randomized to either Blalock-Taussig shunt or right ventricle to pulmonary artery conduit, revealed that of the patients that were discharged home after their procedure without transplant listing 14% achieved the definition of advanced heart failure, including 2/3 that were listed for transplantation and just over ¼ who died, by age 6 years (14). Notably, poor exercise performance in Fontan patients has been associated with increased morbidity and mortality (15) as has poor CHQ-PF50 physical summary score (13).

### Heart Transplant Waitlist

Once listed for heart transplant patients must wait for a donor heart variable amounts of times depending on a number of important variables including listing status, size, sensitization and blood type, among others. The number of children listed for heart transplantation is higher than the number of available donor organs, resulting in increasing waitlist times, particularly for children between 10 and 25 kg in weight and those with O blood type (6). The adoption of ABO incompatible heart transplantation for children <2 years of age resulted in improved wait times for this population with comparable long-term post-transplant outcomes (16, 17). Highly sensitized patients, those with pre-formed anti-HLA antibodies against potential donors, have been noted to have increased post-transplant morbidity and mortality (18). Notably, in the absence of convincing clinical evidence for a preferred strategy, management of these patients varies regarding evaluation and response to existing anti-HLA antibodies, desensitization, exclusion of potential donors, and willingness to transplant against donor-specific pre-existing antibodies. The most prevalent current strategy involves waiting for donors against whom no unacceptable antibodies pre-exist, which often leads to prolonged wait times and potentially increased waitlist mortality (18, 19).

Ventricular assist devices have been increasingly used in children waiting for heart transplantation with resulting reductions in waitlist mortality (20). As technology and clinical expertise has increased with approximately half of pediatric patients currently being on VAD support at the time of transplantation, though the proportion is smaller in single ventricle patients (10). However, there is significant morbidity associated with this invasive mechanical support strategy, most importantly stroke and infections (21). Recent efforts through the ACTION learning network to standardize stroke prevention and anticoagulation in pediatric patients supported with Berlin VAD have demonstrated an extraordinary reduction in the rate of stroke from a baseline of 30% to <12% through a multiinstitutional quality improvement strategy (22). In spite of these improvements this population continues to suffer from high burden of physical and neurologic limitation.

### Pediatric Heart Transplant Outcomes

The overall outcomes for pediatric heart transplant recipients are excellent, with 5-year and 10-year post-transplant survival approaching 80 and 65% respectively. It is notable that outcomes are also improving over time, with those in the most recent era (2010–2017) having 1-year post-transplant survival of ~90% compared to just below 70% for those in the earliest era (1982–1991), owing primarily to improvements in the operative, perioperative and early post-transplant management including improvement in immune suppression (10). Notably hypertension, tremor, diabetes, renal dysfunction, decreased bone density and development of PTLD are common side effects of immune suppression medications that can lead to significant morbidity (23). One approach to ameliorate medication side effects has been the utilization of steroid sparing regiments, relying on induction therapy, with comparable outcomes (24).

A number of other important factors affect median survival including age, rejection, presence of CHD and pre-transplant need for mechanical support, among others. Patients <1 year of age at transplantation have the highest perioperative mortality risk, however they also enjoy the lowest rate of graft loss over time, leading to the longest median survival, currently over 20 years (10). Patients in older age groups have comparable perioperative risk to one another but older children have increasing yearly rates of graft loss leading to progressively shorter median survival rates (10). Children who experience an episode of rejection requiring treatment during the first post-transplant year have been shown to have increased rate of graft loss over time (10). While donor factors are important, including lifestyle factors like smoking and drinking (25) the most important pre-transplant determinants of graft survival are recipient specific such as need for peri-transplant mechanical support, invasive ventilation, hepatic dysfunction and renal replacement (26). Notably, while the use of ECMO is associated with increased (27) pre- and post-transplant mortality the use of VAD demonstrates equivalent post-transplant outcomes to those patients not requiring mechanical support (28). The presence of CHD, as compared to cardiomyopathy, is also associated with decreased waitlist and post-transplant survival, particularly for children aged 5 years old and younger (10). For single ventricle patients, stage of palliation at transplantation is also associated with changes in expected survival, with Fontan patients having the highest survival.

In addition to the evolving improvements in outcomes across the board for pediatric heart transplant patients, >80% of post-transplant patients have minor to no activity restrictions, based on Lansky scores when evaluated at 1, 2, and 3-years post-transplant (10). While these patients are burdened with significant complications, the perception of quality of life among these patients at ten-years post heart transplant is similar to the population at large (29).

## Functional Impact of Heart Failure and Heart Transplant

Generally, when rehabilitation is discussed in patients with heart failure or heart transplant, the focus has mainly been around exercise capacity and exercise training. However, there are other areas of function that might be impacted due to their cardiac function, comorbidities/complications related to heart failure and treatment for heart failure. As mentioned previously the complexity of cases of CHD that get a heart transplant has increased.

Parents of children with CHD commonly report these children experience difficulty with learning, communication, self-care, fine and gross motor skills. Additionally, these children miss more days of school and participate in less extracurricular activities than children with special healthcare needs without heart disease (30). Children with heart failure will have some degree of functional impact and will affect their quality of life throughout their lifespan.

There are four main areas of function: gross motor related to mobility/endurance, fine motor skills and activities of daily living, speech/cognition and feeding. Each of these areas are treated by specific rehabilitation therapists: physical and occupational therapy, speech and language; and feeding. These interventions will be described later in the paper.

Certain complications, comorbidities or secondary effects are related to heart failure and will affect the patient’s function:

### Neurologic

#### Stroke

The most common cause of stroke in children is CHD or the treatment of CHD (31). Abnormal cardiac anatomy and function can increase the risk of thromboembolism. A general prothrombotic state can also be found in CHD as a result of chronic illness, infection, iron deficiency anemia or erythrocytosis, persistent low-grade inflammation, coagulation system abnormalities, abnormal flow patterns and multiple surgical interventions (32).

Some of the procedures used to support heart failure patients also have a high neurologic risk. A prospective cohort study revealed that 29% of patients on Berlin Heat have a at least one neurologic event, with most of them occurring within the first 2 weeks after implant (21). Major efforts are currently being made to decrease the incidence of stroke in VAD patients (22). For patients placed on ECMO incidence of neurologic events was 12%, with longer the duration of ECMO and neonates being risk factors (33). Of note, typical length of support on VAD is months or years, as opposed to days to weeks on ECMO. In adults having a heart transplant in the setting of heart failure decreased the risk for stroke (34).

These neurologic events can affect their muscle tone, their mobility, use of upper extremities, participation in activities of daily living, feeding (35), speech and language (36), cognition and neurodevelopment (37).

#### Neuropathies

Whilst relatively rare these can also happen in patients who undergo heart transplant and it can be related to positioning, ischemia and secondary effects of some of the transplant medication (38, 39). These injuries can result in motor and sensory impairment as well as neuropathic pain depending on the nerve and severity of the injury.

#### Neurodevelopmental Delays and Functional Impact From CHD

As we have previously mentioned an important number of pediatric cardiac transplants are complex CHD patients. There has been extensive literature regarding neurodevelopmental delays in patients with CHD with abnormal executive function, cognitive and communication difficulties with widely implemented management guidelines (40). There are also descriptions of the acute functional impact of cardiac surgery on CHD patients, with almost half of patients who undergo cardiac surgery on cardiopulmonary bypass requiring rehabilitation therapies in the acute post-operative setting (41). Parents of children with congenital heart disease commonly report these children experience difficulty with learning, communication, self-care, fine and gross motor skills. Additionally, these children miss more days of school and participate in less extracurricular activities than children with special healthcare needs without heart disease (42).

### Decreased Exercise Capacity

Exercise capacity is decreased in patients with heart failure (43), including those supported on VADs (44). Similar findings exist for patients after heart transplant (45). This decreased exercise capacity can significantly affect their quality of life.

Peak oxygen consumption, the gold standard for exercise capacity, has been showed to be related to morbidity and mortality in children with various forms of CHD (46–48) and in adults following heart transplantation. It indicates an inability of the cardiopulmonary system to meet the increased metabolic demands of physical activity. Predicted Peak VO2 ranged from 25% to 43% in the immediate posttransplant period to 57–73% at 0.6–20 years. In a cohort of pediatric patients with VADs PVO2 was 19 ± 6.3 ml/kg/min (44).

In heart transplant patients this exercise limitation is mostly due to the cardiac autonomic denervation that can lead to chronotropic impairment (49) due to the loss of sympathetic and parasympathetic innervation of the atrial node. Abnormal vascular and muscular function (50) due to deconditioning and secondary effects of immunosuppression treatment (51–53) can also affect exercise capacity. Sometimes, the limitation is due to impaired stroke volume (45). In patients with VADs exercise limitation is due to the inability to increase their stroke volume (44) to meet increased metabolic demands.

Some of these limiting factors can improve with exercise training such as deconditioning and vascular function. Over the past few years there has been an increase in studies on exercise training in patients with CHD, many have shown improvement in exercise capacity (54, 55). Research in exercise training in pediatric heart transplants (56, 57) is more limited but has shown promising results. In adult transplant patients exercise training has been shown to improve strength and exercise capacity (58), decrease readmission risk (59) and increased long term survival (60). Data on programs for patients with VAD is sparse (44, 61). It has been shown to be a safe intervention in all of these patient populations.

### Feeding and Speech

#### Feeding

Dysphagia or feeding difficulties can entail difficulty with the oral preparatory phase of swallowing (chewing and preparing the food), the oral phase (moving the food or fluid posteriorly through the oral cavity with the tongue, into the back of the throat) and the pharyngeal phase (swallowing the food or fluid and moving it through the pharynx to the esophagus). The different parts of the process can be affected in patients who undergo heart transplant and specially those who had a baseline CHD diagnosis (62). Feeding difficulties are multifactorial and incompletely understood in the CHD population (63, 64).

Risk factors for developing feeding difficulties in these patients include neonatal age (65), use of TEE, prolonged intubation, having a stroke or hypoxic ischemic encephalopathy, a vocal cord paralysis/palsy, prolonged ECMO course and a low birthweight (30, 66–68).

Complications of dysphagia have been shown to include aspiration leading to pneumonia, respiratory arrest, progressive chronic lung disease, malnutrition, increased risk of infection, prolonged length of stay in hospital, and increased risk of death (35, 69, 70). Therefore, early identification and treatment is crucial for this patient population.

#### Speech and Language/Cognition

Communication and cognition can be affected due to multiple reasons in patients with heart failure and transplant. Some of the causes are shared with the feeding difficulties such as injury to the recurrent laryngeal nerve with vocal cord paralysis/palsy (66), prolonged intubation with laryngeal and vocal cord injury (71) or stroke (32).

In the acute hospital setting some patients may be non-verbal due to tracheostomy, mechanical ventilation and intubation (72). Providing these patients with alternate forms of communication may allow them to communicate more effectively with the medical team and family might improve outcomes and increase their participation in their rehabilitation process (73).

## Rehabilitation Programs

The goal of Rehabilitation is to enhance and restore functional ability and quality of life to those with physical impairments or disabilities that can be temporary or permanent. To do so a personalized program is developed depending on the needs of the specific patient.

A Rehabilitation program can include medical management, use of rehabilitation equipment as well as the involvement of a variety of rehabilitation therapists that include physical, occupational, speech and feeding therapists.

Recommendations for the different types of programs is based on recommendations from rehabilitation medicine, the cardiac surgical and medical teams, and the rehabilitation therapists both in the in- and outpatient settings.

These programs and therapies can be provided in different settings:

- Outpatient clinics for patients prior to surgery or that have discharged home.
- Acute inpatient hospital setting for those patients admitted for medical or surgical management of their heart condition.
- Acute inpatient rehabilitation for patients with major functional impairments, who require ongoing acute medical management, intensive rehabilitation therapies, education for families, rehabilitation equipment evaluations and help with the supports at home and in the community.
- School and Early Intervention.

Rehabilitation programs include the following critical components:

### Physical Therapy and Occupational Therapy

Physical Therapists and Occupational Therapists play a vital role in the life of a child with heart failure. Children with heart failure are often referred to physical and occupational therapy for treatment of developmental delays (74). Children with congenital heart disease are at risk for early developmental delays, development of cognitive dysfunction, impacts on quality of life, delays in speech and language, deficits in attention, visuospatial, and executive functioning as well as emotional behavioral dysregulation (75). Heart transplant in infancy and toddlerhood has been associated with mild delays in motor and cognitive development (76).

#### Evaluation Process for Physical and Occupational Therapy

Physical and occupational therapy evaluations begin with a thorough chart review to understand the child’s diagnosis, medical status and prognosis. The therapist will have an understanding of basic lab values and how this may impact evaluation and intervention as well as vital sign parameters for activity. The therapist should collect a developmental history from the patient and caregiver along with prior level of function, previous need for therapy services as well as home set up and caregiver support. It is also important to know if the child uses an assistive device or bracing at baseline.

#### Physical Therapy

Children with heart failure often require physical therapy in a variety of settings to improve exercise capacity and endurance, progress gross motor skills, restore function and improve quality of life. Many children with heart failure may go on to be listed for heart transplantation, therefore physical therapy plays an important role to optimize the child’s endurance and function prior to heart transplantation. Physical therapists will conduct an examination, collaborate with patients and families to create goals and establish a plan of care for children with heart failure.

The physical therapy examination will assess the child’s impairments, activity limitations and participation restrictions which will vary based on the child’s age, prior level of function and current medical status. The physical therapy examination may include assessment of muscle strength, joint range of motion, sensory and proprioceptive testing, coordination, balance, endurance, functional mobility, gross motor skills and developmental skills.

The use of standardized assessments is recommended during a physical therapy examination in order to obtain a quantifiable measurement of a child’s baseline and enable measurable goals to be set. Repeat use of standardized assessments at re-evaluation will provide valuable data on the child’s progress. Infants and toddlers with heart failure often present with delayed attainment of gross motor milestones due to prolonged hospitalizations, previous surgeries, decreased strength and endurance. Children and adolescents with heart failure often present with decreased exercise capacity and may also present with strength, balance and coordination impairments due to pharmacological treatment causing neuropathies as well as sedentary lifestyle. Selection of a standardized assessment is made based on the child’s age and specific areas of impairment or skills that have been identified as areas of concerns by parents and providers.

The 6 min walk test is easily performed, widely available and a well-tolerated test for assessing the functional capacity of patients with heart failure in everyday practice (77). The 6-min walk test has also been shown to have predictive value for mortality or heart transplantation in children with dilated cardiomyopathy who are 6 years of age or older for mortality or heart transplantation (78). The Bruininks Oseretsky Test of Motor Proficiency-2 (BOT-2) (79) can be utilized in children and young adults ages 4–21 years of age to assess stability, mobility, strength, coordination and object manipulation. The Test of Gross Motor Development 3 (TMGD-3) (80) is a norm referenced tool used to identify children with gross motor deficits and is valid for children ages 3–11. The TGMD-3 is made up of two subtests, locomotor and object control, encompassing 12 skills and can be administered in 15–20 min. This may be appropriate to assess for gross motor deficits in children with limited tolerance to activity. For the younger populations, the Alberta Infant Motor Scale (AIMS) (0–18 months) (81), Test of Infant Motor Performance (TIMP) (82) (34 weeks post conceptual age to 4 months) and Peabody Developmental Motor Scales 2 (PDMS-2) (83) (0–5 years of age) are useful tools to assess gross and fine motor skills, identify developmental delay and assign age equivalences when appropriate. The TIMP has been widely used in the congenital heart disease population to assess neuromotor performance before and after open heart surgery (84–86). In the inpatient setting, limitations in completion of standardized testing may exist due to presence of lines and tubes, post-surgical precautions, or limited tolerance and endurance.

For quality-of-life assessments the PedsQL and CHQ-PF50 have been used in the pediatric cardiac population (13, 87).

Following an evaluation, the physical therapist will synthesize the information gathered to create measurable goals with the patient and caregivers and establish a plan of care individualized for each child. Typical goals for infants and toddlers with heart failure are to progress gross motor skills. Goals for children and adolescents will vary widely based on evaluative findings but may include improving strength, balance, coordination, endurance and functional mobility.

An individualized physical therapy plan of care is created for each child and frequency of services is established. Physical therapy intervention is especially important for children with heart failure awaiting heart transplantation. Adult literature has shown that physical rehabilitation can improve functional capacity and quality of life, decrease hospitalizations and improve mortality in adults with heart failure (88, 89). Literature on its effect in the pediatric population is limited. A 2007 study by McBride and colleagues establishes that inpatient exercise programs for children awaiting heart failure are safe and feasible (90). Patients and caregivers will be educated on progression of activity or developmental skills, energy conservation techniques and postsurgical sternal precautions when applicable.

Therapeutic interventions may vary depending on the inpatient or outpatient setting and availability of resources. Interventions will include facilitation of gross motor skills, strength training, balance and coordination training and endurance training. The physical therapist will modify activity based on the child’s age, oftentimes incorporating age-appropriate play to help engage the child.

#### Occupational Therapy

Occupational therapists play an important role for patients with heart failure. Occupational therapists are clinicians trained to work with heart failure patients across the lifespan. Occupational therapists are able to optimize the quality of life and help restore function (91). The main areas of care with these patients include occupation centered goals, caregiver’s involvement, facilitation of social participation, and improving quality of life. “In occupational therapy, occupations refer to the everyday activities that people do as individuals, in families, and with communities to occupy time and bring meaning and purpose to life. Occupations include things people need to, want to and are expected to do” (92). A person’s occupations include activities of daily living (ADLs), instrumental activities of daily living (IADLs), health management, rest and sleep, education, work, play, leisure, and social participation (93). All areas of occupations can be impacted as one experiences heart failure throughout their life.

Occupational therapy evaluations will focus on a variety of performance skills and client factors including cognition, strength, ROM, coordination, development, balance, functional mobility and ability to participate in ADLs and play. An occupational therapist may complete of standardized and non-standardized tests as determined by the needs of the child and family, the identified impairments the setting in which the evaluation takes place. The use of standardized assessments assists in establishing a baseline of occupational performance which allows for objective measurement of progress after and during intervention.

Following an evaluation, the occupational therapist will develop an individualized plan of care that includes measurable goals that are developed with the patient and caregivers. Typical goals for infants and toddlers with heart failure are to progress their developmental skills and provide education to the caregivers for carryover. Goals for children and adolescents will vary widely based on the findings but may include improving functional strength and endurance, coordination, cognitive retraining, visual motor skills, energy conservation techniques, modifications for activities of daily living skills and coping strategies. Occupational therapy interventions support patient’s recovery and provide direct therapy to overcome any barriers that prevent them from participating in their occupations.

#### Ventricular Assist Devices

In some instances, children with heart failure may require advanced support and implantation of ventricular assist devices. Post surgically, physical therapists and occupational therapists play a role in early mobility and restoring function. Postoperative precautions may be present following implantation of ventricular assist devices including sternal precautions, presence of multiple lines and tubes and vital sign parameters. Communication with the medical team is vital to determine timing of early mobility, specific precautions and vital sign parameters particularly during mobility and ADLs. Additionally, therapists play an important role in educating patients and families about postoperative precautions and adjustment to daily activity with their new device. This often includes education on sternal precautions, drive line precautions, connecting the VAD to battery power, and donning and doffing their VAD carrying device. As discussed above, there is also a high risk of neurologic complications associated with ventricular assist devices. Physical therapists and occupational therapists will be vital in assessing for neurologic compromise and treating impairments, activity limitations and participation restrictions to restore function post operatively. Following the immediate post-operative period, physical therapy is often indicated for continued strength and endurance training in preparation for heart transplantation. Occupational therapy is vital to address orthotic and/or rehabilitation assistive devices as well as adaptations to complete ADLs and engage in play based and school activities while inpatient. For the neurodevelopmental population, optimizing gross and fine motor skills prior to transplant is the focus of physical and occupational therapy. This often involves creative strategies and adaptations to activities to accommodate the patient’s VAD and maintain safety and precautions.

### Exercise Training

In adults, there is evidence of improved exercise capacity after cardiac rehabilitation in patients following heart transplantation (58). In adults with heart failure these rehabilitation programs reduce the risk of all-cause hospital admissions, as well as reduce HF-specific hospital admissions in the short term. Cardiac rehabilitation may confer a clinically important improvement in health-related quality of life (94). These interventions have been shown to be safe. The guidelines for cardiac rehabilitation are well established (95–97) and there tends to be agreement amongst countries (98).

However, in pediatrics evidence regarding the effects of cardiac rehabilitation programs is growing but has shown promising results (54). Evidence is even scarcer for heart transplant patients (56, 57) and patients on VADs (61). Exercise training in the pediatric cardiac population also appears to be safe. Guidelines for exercise training in the pediatric cardiac population have not yet been established but there is an increasing interest in creating them.

We would like to highlight that exercise training in pediatric patients with heart failure or transplantation might take place years after their surgery, due to their ability to participate in formal exercise testing and a structured exercise program. This is different to adults who normally receive cardiac rehabilitation after a surgical intervention.

Based on literature mostly focused on CHD and adults, as well as clinical experience we would recommend the following program:

#### Pre and Post-program Evaluation

A cardiopulmonary exercise test (CPET) prior to the program is recommended to better understand the factors contributing to a lower exercise capacity. A CPET will also help ensure the safety of initiating a rehabilitation program and help structure the exercise training component. Post-program it can help quantify the impact of the program.

#### Exercise Training Program

##### Prescription

The patient will be provided with an individualized exercise prescription based on the CPET, diagnostic testing, comorbidities and goals of the patient. This prescription will follow the FITT principle of exercise frequency, intensity, time, type, volume and progression.

##### Location

Centered based programs last ~12 weeks, with ideally two supervised exercise sessions per week. The program should be over seen by a medical provider. Some of the studies have been homebased.

##### Program Components

- Aerobic training: should be the main component of the program. Intensity can be established by the Borg Scale, the Talk test and the heart rate reserve. However, the heart rate reserve might not be applicable to the heart transplant patients.
- Resistance training: include exercises with low resistance and a high number of repetitions.
- Warm up and cool down exercises.
- Education: regarding cardiac diagnosis, CV risk factors, physical activity and nutrition.

##### Monitoring

*Vitals Signs*

Blood pressure, oxygen saturation and heart rate should be taken before and after the program.

*Telemetry*

Depending on clinical recommendations.

##### Precautions

- Initiate further assessment of the patient if symptoms such as dizziness especially if accompanied by drop in BP, concerning arrhythmias, or chest pain or dyspnea felt to be cardiac in nature appear.
- If on Telemetry: Exercise to be stopped if patient has clinically significant STT wave changes, sustained ventricular tachycardia, high grade ectopy or AV block.

### Feeding and Swallowing: Assessment and Management

Patients with VAD and recovering from heart transplant are at significant risk of dysphagia (as described above) and timely assessment of feeding skills and swallow function is crucial. A speech-language pathologist (SLP) specializing in feeding and swallowing should conduct a clinical assessment of feeding and swallowing should as soon as the patient is deemed clinically ready to consider oral feeding. In determining readiness for assessment, the patient’s tolerance of enteral feeds, degree of respiratory support and level of alertness should be considered. In general, patients should demonstrate adequate tolerance of gastric feeds prior to initiation of oral feeds as feeding intolerance may impact both the desire to eat, as well as the safety. Given increased risk of aspiration while on NIPPV (99) and post-extubation (100–102), consideration of oral feeds should occur once a patient is tolerating breathing on room air or low flow nasal cannula only. With regards to level or alertness and behavioral state, patients should be able to maintain a calm, alert state for the length of a typical mealtime. In addition, the patient needs to tolerate being in a typical feeding position for age (i.e., semi-upright or sidelying for infants, sitting upright for older children).

Clinical assessment of feeding and swallowing should consist of thorough history gathering including baseline feeding difficulties, risk factors for dysphagia, assessment of state and baseline status, oral mechanism examination, assessment of vocal quality, assessment of non-nutritive suck and oral reflexes in infants, all prior to offering any oral trials. When offering oral trials, clinicians should proceed slowly and with caution. In infants, a very slow flow nipple should be utilized, in particular if risk factors for aspiration from above have been identified. In older children, oral trials should begin with ice chips or water via teaspoon, proceeding to larger bolus size and consecutive swallows dependent on if the patient is demonstrating any overt signs of aspiration or signs of stress. Depending on state and degree of deconditioning, small volume trials of purees and developmentally appropriate solid foods can also be considered. When offering solid foods, chewing skills, ability to fully pulverize solids prior to swallowing and oral clearance should be considered in determining safety for solids and choking risk. Oral trials should be discontinued at any point where the patient is demonstrating overt signs or aspiration or physiologic instability.

The primary focus of a clinical feeding and swallowing evaluation should be on safety to proceed with oral trials, including safe oral diet recommendation and need for further instrumental assessment of swallow function. Additionally, a patient’s interest in oral feeding and developmental feeding skills should be taken into consideration. Patients should not be pushed to participate in assessment if they are resistant, nor if they are not alert enough to actively engage.

Many patients following heart transplantation will require instrumental assessment of swallow function. Instrumental assessment of swallow function should be considered if there are overt signs of aspiration on clinical assessment, or if the patient is considered at high risk of aspiration based on history, in particular patients with neurologic insult (103, 104) or known or suspected vocal fold immobility (62, 105). Timing of assessment should be carefully considered, taking into account level of alertness, sedative medications, need for respiratory support and likely trajectory of improvement. Depending on the patient’s age, willingness to participate, level of medical acuity and mobility level, videofluoroscopic swallow study (VFSS) or fiberoptic endoscopic evaluation of swallowing (FEES) can be considered. There are advantages and disadvantages to each exam and determining which type of assessment to pursue should be discussed with the patient’s full medical team (106, 107). In patients with VAD, in particular, risks and benefits of instrumental assessment need to be carefully weighed.

Management of dysphagia in pediatric patients with VAD or following heart transplant will occur based on findings from clinical and instrumental assessments of feeding and swallowing. In patients found to aspirate on instrumental assessment, strategies to improve airway protection from above should be trialed during the instrumental assessment to determine safety and improvement. These strategies may include altering positioning, altering method of delivery of liquids (e.g., slower flow nipple, straw cup, valved sippy cup), pacing bottle feeding or adjusting bite size or food consistency, and thickening liquids. Decisions regarding use of thickened liquids should be guided by instrumental assessment of swallow function whenever possible. Given increased incidence of silent aspiration in patients with vocal fold immobility or neurologic insult, as well as challenges with thickening liquids in the pediatric population as a whole (108), use of empiric thickening is discouraged. Usage of thickening agents should be discussed with medical team prior to initiation, with consideration of patient’s age and gastrointestinal history. In some instances, patients are found to aspirate all consistencies on instrumental assessment of swallow function, despite use of therapeutic strategies, and in these cases, alternative means of non-oral nutrition and hydration are necessary.

Once a patient’s swallowing safety has been established via clinical and instrumental assessment as appropriate, while inpatient, feeding therapy should be provided with goals of monitoring safety with oral feeds, and advancing oral intake as tolerated. Clinicians should be closely monitoring swallow function and signs of aspiration with oral trials, and making adjustments to positioning, delivery method and diet recommendations as appropriate. In addition, goals such as reducing oral hypersensitivity and improving tolerance of oral stimulation, as well as improving oral motor skills for feeding can be addressed. In young patients who are deemed unsafe for oral feeding, a plan for oral stimulation should be provided to avoid development or worsening of oral aversion, as well as to promote ongoing advancement of oral motor skills for feeding. Oral stimulation should be developmentally appropriate and may include use of pacifier, safe teething toys, familiarization with feeding utensils and therapeutic tastes. Principles of motor learning should be kept in mind when treating both infants who are learning to swallow and in retraining swallowing with older children (109). Parent education and training are essential components of any feeding therapy provided to infants and young children (110). Patients with a history of congenital heart disease have increased incidence of baseline feeding and swallowing difficulties (111, 112) and may require extensive feeding therapy, extending beyond the inpatient admission, to target acceptance of oral feeds and oral motor skills. Inpatient clinicians should ensure that referrals for feeding therapy through rehab or in the outpatient setting are made.

Need for supplemental nutrition should be closely monitored to ensure that opportunities for oral intake are maximized as is safe, while nutrition and hydration needs are met (113). Most patients will require at least temporary alternate means of nutrition and hydration as their strength improves and swallow function is assessed. The medical team should closely monitor tolerance of gastric feeds and may determine that the patient requires post-pyloric or parenteral nutrition. For the most part, patients need to be tolerating gastric feeds to begin working toward full oral feeding. Many patients will require longer term non-oral means of nutrition if they continue to be deemed unsafe to feed by mouth from a swallowing standpoint, do not have the endurance to maintain full oral intake over time, or have oral aversion and are working on acceptance of oral trials. Placement of gastrostomy tubes does appear to be safe following pediatric heart transplant (114), though need for long term supplemental nutrition needs to be carefully considered on a case by case basis. In particular, in patients who have not established oral feeding prior to heart failure or heart transplant, earlier consideration of gastrostomy tube placement may be indicated so as not to prolong length of stay while working toward full oral feeding.

Thus, far there has been limited research regarding the incidence and risk factors for dysphagia in patients with VAD and following heart transplantation, and further study is indicated. Further research regarding efficacy of strategies used to treat dysphagia in this population are warranted. Long-term feeding outcomes for patients who undergo early heart transplantation should also be followed.

### Speech Language, and Communication Therapy

Children with heart failure may present with a broad spectrum of speech, language, and communication needs. Given potential baseline speech and language delays for patients with congenital heart disease (115, 116), heightened risk for neurological complication (21, 32, 36), and risk of communication vulnerability related to medical or surgical intervention, a speech-language pathologist may be an important part of a child’s multi-disciplinary team. Depending on the patient’s baseline skills, current skills, and a variety of variables related to medical intervention and care, the goals of service delivery by the SLP can be multifactorial to include provision of developmental speech, language, and cognitive support, rehabilitation of speech, language, or cognitive-communication skills, and feature-matched assessment to promote functional communication with providers, loved ones, and peers including potential implementation of augmentative and alternative communication (AAC).

To best support speech, language, and communication in children with heart failure, the SLP may perform an initial assessment or screening tailored to each child’s individual needs, age, medical status, setting, and situation. Assessment may include clinical observation, parent interview, language sampling, standardized testing, or criterion referenced measures. Given potential for risk of communication impairment in children with heart failure across a variety of domains, initial screening or assessment should include information gathering and direct observation regarding hearing and vision status, vocal cord function, language comprehension, social-emotional functioning, articulation, language production, literacy, cognition, sensory skills and needs, and motor profile. An oral mechanism exam should also be conducted. Following screening or assessment, the SLP will work with patients and families to determine individualized goals of care. Treatment recommendations may change depending on the child’s medical status, setting, and areas of need.

In many instances, children with heart failure experience prolonged hospitalizations due to medical complexity, surgical interventions, VAD placement, or while awaiting heart transplant. For patients with baseline speech and language impairments prior to their hospitalization, carryover of intervention in the acute care environment may be warranted to support development of skills and to prevent regression, particularly in the context of a prolonged admission. For children with acute changes in their speech, language, or communication profile, early intervention in the acute care setting is critical to support communication access, early rehabilitation, and ongoing monitoring of clinical status. As many children with heart failure have extended hospital stays while waiting for transplant, possibly following an acute neurological event, vocal cord injury, or sensory impairment (e.g., vision or hearing), their access to rehabilitation outside of the acute hospital setting can be limited. Therefore, targeted intervention to support speech, language, and cognitive communication should be implemented early for optimal outcomes.

Patients with heart failure may experience acute functional communication difficulties or a non-speaking status secondary to new communication impairments (e.g., neurologic injury, prolonged intubation, delirium, vocal cord injury, tracheostomy), or due to baseline communication impairments. This inability to functionally communicate puts patients at heightened risk of sentinel and adverse events, increased length of stay, and can impact psychological well-being (117). Regarding patients with heart failure or following heart transplant, an inability to functionally communicate can impede their ability to participate in other rehabilitation activities, which can hinder progress. For patients with an acute non-speaking status or with a functional communication difficulty, implementation of augmentative and alternative communication (AAC) should be considered to support a reliable way to effectively communicate (118). The SLP should conduct a feature-matched assessment to match the patient’s skills, strengths, and needs to available tools and strategies (119). Interventions may include aided (involving use of external materials such as writing tablets, communication boards, speech-generating devices, etc.) and unaided (involving use of one’s own body such as gestures, facial expressions, eye gaze, blinks, etc.) strategies (120). AAC interventions to support functional communication needs may change frequently depending on patient status, needs, and preferences.

For ongoing speech, language, and communication needs following heart transplant or discharge from the acute care setting, continued intervention may be warranted to support functional communication, academic participation, social experiences, and improve quality of life. Ongoing interventions and recommendations should be highly individualized to each child’s specific needs based on findings from comprehensive and dynamic assessment. Goals may be supportive in targeting areas of need across a variety of communicative domains, including expressive and receptive language, articulation, voice and resonance, cognitive communication, literacy, and pragmatics. For children who cannot functionally access spoken language, implementation of AAC tools and strategies may be required for ongoing communication access needs. Treatment goals and recommendations, including areas targeted and frequency of intervention, may change over time to meet evolving needs. Changes in health status may also impact treatment approaches or recommendations within this population.

### Limitations of Rehabilitation Therapies in the Acute Inpatient Hospital Setting

The acute care setting often poses challenges when completing evaluations and providing direct interventions for all rehabilitation therapists. These challenges are not limited to medical instability, limits within the physical setting and time constraints due to multiple medical providers and treatments. Patients with heart failure are often attached to various medical machines, lines and tubes that may require additional staff for safety and monitoring of hemodynamic status during mobility, ADLs, feeding and complicating communication. Many medications used to treat heart failure may impact patients’ level of arousal and ability to cognitively participate in their therapy sessions. Infants often required prolonged hospitalizations during critical periods of development. There is a challenge in the acute care setting to normalize an infant’s environment in a way that allows for them to progress their developmental milestones. Children and adolescents who require prolonged hospitalizations have interruptions in their daily routines and it is difficult to simulate day to day activities and routines within the acute care environment.

There is limited research in the pediatric setting on appropriate exercise guidelines and vital sign parameters particularly for children with very low ejection fraction. Additionally, children with heart failure often exhibit growth failure due to high metabolic demands and increased energy expenditure, which can lead to failure to thrive and further medical complications (121). Discussion with the medical team is imperative to determine the appropriate precautions, vital sign parameters and exercise guidelines. Future research should be directed at determining activity guidelines and safe vital sign parameters for children with heart failure.

## Conclusion

Survival has increased in children with heart failure, including those necessitating heart transplantations, due to advances in medical and surgical management. However, quality of life and function might be compromised due to the comorbidities and complications of heart failure and its treatment.

It is essential to provide heart failure patients with individualized multidisciplinary rehabilitation interventions both in the in- and outpatient settings to optimize their function and quality of life.

## Author Contributions

AU contributed to conception and design and writing of the paper. EB, EL, KE, MS, and PE wrote sections of the manuscript. PE overviewed the paper. All authors contributed to manuscript revision, read, and approved the submitted version.

## Conflict of Interest

The authors declare that the research was conducted in the absence of any commercial or financial relationships that could be construed as a potential conflict of interest.
